# Effects of non-pharmacological interventions on anxiety, depression, and sleep quality in patients with postoperative glaucoma: A protocol for systematic review and network meta-analysis

**DOI:** 10.1097/MD.0000000000027090

**Published:** 2021-09-03

**Authors:** Jiong Liu, Lei Cao, Guang Yang, Runhai Zhou

**Affiliations:** a Department of Ophthalmology, Beijing Jishuitan Hospital, Beijing, China; b Department of Ophthalmology, General Hospital of North Theater Command, Shenyang, Liaoning Province, China.

**Keywords:** anxiety, depression, glaucoma, meta-analysis, non-pharmacological intervention, protocol, sleep quality

## Abstract

**Background:**

Glaucoma is the second most-common blinding ophthalmic disease in the world, and its incidence has been rising year by year in recent years. Currently, the main treatment of glaucoma still relies on surgery. Glaucoma patients often suffer from various psychological problems like anxiety and depression not only because of the lack of understanding of the surgical treatment of glaucoma, but also the long-term stress and the poor prognosis. As alternative therapies, non-pharmacological interventions can greatly alleviate psychological burdens and improve sleep quality in surgically treated glaucoma patients. Randomized controlled trials of non-pharmacologic interventions for glaucoma have been reported, although the results remain conflicting. Evidences for determining the efficacy of non-pharmacologic interventions for glaucoma are scant. This study aims to assess the effects of non-pharmacological interventions on anxiety, depression, and sleep quality in patients with postoperative glaucoma through a network meta-analysis.

**Methods:**

A systematic search of relevant literatures published before August 2021 about the effects of non-pharmacological interventions on anxiety, depression, and sleep quality in patients with postoperative glaucoma will be performed in Wanfang, VP Information Chinese Journal Service Platform, China National Knowledge Infrastructure, Chinese BioMedicine Literature Database, Pubmed, Embase, Cochrane, and Web of science. Two reviewers will be independently responsible for literature screening and selection, quality assessment, and data extraction. WinBUGS 1.4 will be used for the network meta-analysis.

**Results:**

This meta-analysis will provide additional and stronger evidences for non-pharmacological interventions on anxiety, depression, and sleep quality in patients with postoperative glaucoma, which will help clinicians and decision makers to make an optimal therapeutic strategy.

**Conclusion:**

This study will provide a reliable evidence-based basis for the clinical application of non-pharmacological interventions on anxiety, depression, and sleep quality in patients with postoperative glaucoma.

**Ethics and dissemination:**

Ethical approval was not required for this study. The systematic review will be published in a peer-reviewed journal, presented at conferences, and shared on social media platforms. This review would be disseminated in a peer-reviewed journal or conference presentations.

**OSF registration number:**

DOI 10.17605/OSF.IO/TYJPK.

## 1 INTRODUCTION

Glaucoma is a disease characterized by optic nerve atrophy and visual field defects, which reduces visual acuity and effective visual field.^[1–3]^ It is the second most-common blinding eye disease in the world, which has become an important threat to human visual health that seriously affects daily life and work of affected people.^[4,5]^

Surgery is currently an effective clinical ophthalmic treatment of glaucoma with a moderate efficacy. Glaucoma poses a huge impact on the vision, and most of them have varying degrees of anxiety and depression.^[6–8]^ In addition, the lack of understanding of the surgery makes glaucoma patients become more worried, anxious, and depressed during the treatment, which ultimately influence the clinical outcome. It is reported that approximately 50% of glaucoma patients suffer a more severe anxiety and depression psychological state.^[9]^ Anxiety and depression are key factors affecting the prognosis of glaucoma.^[10–12]^ In addition, anxiety and depression can cause somatic reactions like increased intraocular pressure, sleep disturbances, and abnormal blood pressure in glaucoma patients.^[13,14]^ Therefore, how to alleviate anxiety and depression and improve sleep quality in patients with postoperative glaucoma is a clinical issue worth exploring.

Currently, pharmacological and non-pharmacological interventions are the main therapeutic strategies for anxiety, depression, and sleep problems. Considering the adverse events and economic costs of pharmacological interventions, non-pharmacological interventions have been well concerned and widely explored. A meta-analysis conducted by Lin et al showed that bright light therapy improves depressive symptoms and sleep disturbances in patients with Parkinson's disease.^[15]^ Yang et al reported that Badaunjin combined with auricular pressure therapy improves sleep quality, anxiety, and depression symptoms in patients with coronavirus disease 2019 insomnia.^[16]^ Tsai et al found that aromatherapy improves anxiety, depression, and sleep-related health problems in menopausal women.^[17]^

Non-pharmacological interventions may be effective interventions for alleviating anxiety, depression, and sleep disturbances in patients with postoperative glaucoma, including positive meditation, muscle relaxation, cognitive behavioral therapy, and acupuncture.^[18–24]^ To date, the effects of different non-pharmacological interventions on anxiety, depression, and sleep quality in patients with postoperative glaucoma remain inconclusive. In addition, few studies have compared the effects of different non-pharmacological interventions. Therefore, it is unclear which non-pharmacological interventions are the most effective in improving anxiety, depression, and sleep quality in patients with postoperative glaucoma.

A network meta-analysis is a tool that can compare and pool evidences from multiple interventions, which also provides relative rankings of clinical outcomes by these interventions.^[25,26]^ We aim to design a new protocol to collect randomized controlled trials (RCTs) of the effects of non-pharmacological interventions on improving anxiety, depression, and sleep quality in patients with postoperative glaucoma through the network meta-analysis. We hope that this study will provide guidance on performing non-pharmacological interventions to improve anxiety, depression, and sleep quality in patients with postoperative glaucoma.

## 2 METHODS

### 2.1 Study registration

The protocol of this review was registered in OSF (OSF registration number: DOI 10.17605/OSF.IO/TYJPK). It is reported to follow the statement guidelines of preferred reporting items for systematic reviews and meta-analyses protocol.^[27]^

### 2.2 Inclusion criteria for study selection

i) Types of studies: RCTs of the effects of non-pharmacological interventions on anxiety, depression, and sleep quality in patients with postoperative glaucoma.
ii) Types of participants: Patients diagnosed with glaucoma. Simultaneous surgical treatment has been performed.
iii) Interventions: The intervention group received non-pharmacological intervention programs, such as psychological interventions, acupressure, acupuncture therapy, exercise therapy, etc. The control group received conventional treatments.
iv) Outcome indicators: Any rating scale that describes anxiety, depression, and sleep quality.

### 2.3 Exclusion criteria

i) Duplicated literatures;
ii) Protocols, case reports, reviews, meta-analyses, conference abstracts, and animal experiments;
iii) Studies without sufficient data.

### 2.4 Data sources

We will systematically search RCTs published in English and Chinese before August 2021 in Wanfang, VP Information Chinese Journal Service Platform, China National Knowledge Infrastructure, Chinese BioMedicine Literature Database, Pubmed, Embase, Cochrane, and Web of science. In addition, the reference lists of the included systematic reviews with meta-analyses will be examined to avoid missing data.

### 2.5 Searching strategy

Literature searching will be conducted using a combination of MeSH terms and free words. The details of search strategies in PubMed were illustrated in Table 1. A similar search strategy will be applied in other online databases.

**Table 1:** Search strategy in PubMed database.

Number	Search terms
#1	Glaucoma[MeSH]
#2	Glaucomas[Title/Abstract]
#3	OR/1–2
#4	Anxiety[MeSH]
#5	Hypervigilance[Title/Abstract]
#6	Nervousness[Title/Abstract]
#7	Anxieties[Title/Abstract]
#8	Depression[MeSH]
#9	Depressive Symptoms[Title/Abstract]
#10	Emotional Depression[Title/Abstract]
#11	Depression, Emotional[Title/Abstract]
#12	Depressions[Title/Abstract]
#13	Depressions, Emotional[Title/Abstract]
#14	Depressive Symptom[Title/Abstract]
#15	Emotional Depressions[Title/Abstract]
#16	Symptom, Depressive[Title/Abstract]
#17	Symptoms, Depressive[Title/Abstract]
#18	Sleep[MeSH]
#19	Sleep, Slow-Wave[Title/Abstract]
#20	Sleep, Slow Wave[Title/Abstract]
#21	Slow-Wave Sleep[Title/Abstract]
#22	OR/4–21
#23	Randomized Controlled Trials as Topic[MeSH]
#24	Clinical Trials, Randomized[Title/Abstract]
#25	Controlled Clinical Trials, Randomized[Title/Abstract]
#26	Trials, Randomized Clinical[Title/Abstract]
#27	Random^∗^[Title/Abstract]
#28	OR/23–37
#29	#3 AND #22 AND #28

### 2.6 Data collection and analysis

#### 2.6.1 Literature screening and data extraction

**Figure 1:**
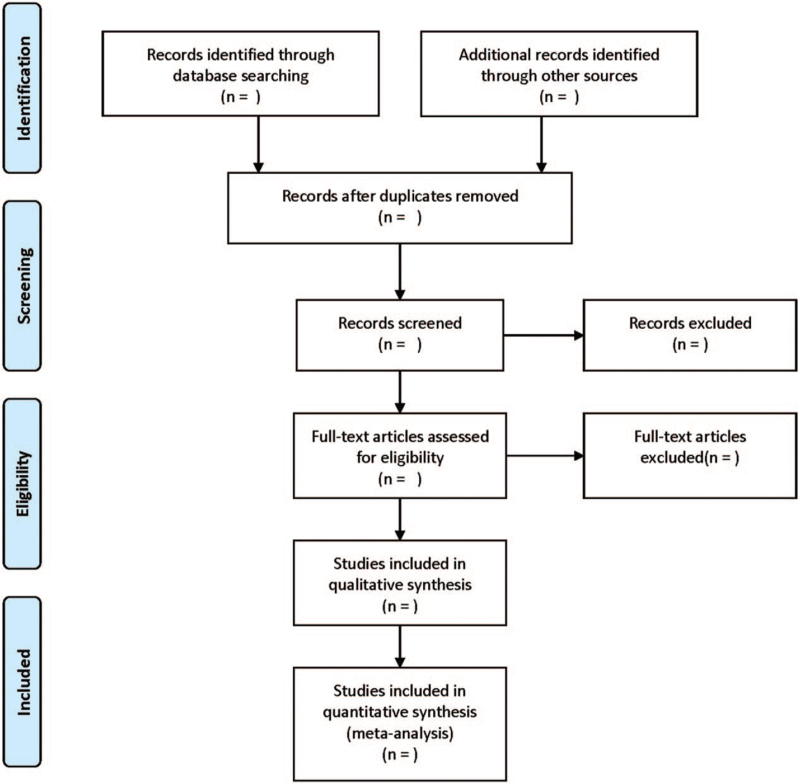
Flow diagram of study selection process.

Literature screening and data extraction will be conducted independently by 2 researchers and crosschecked. Any disagreement will be solved by a third researcher. The items of the data collection table mainly include: first author, publication year, sample size, gender, age, course of disease, intervention measures, course of treatment, and outcome indicators. The screening flow chart of this study was demonstrated in Figure 1.

#### 2.6.2 Assessment of evidence quality

The Cochrane risk-of-bias tool will be used to assess the quality of the included RCTs.^[28]^ This tool examines 6 major domains of bias, including the selection bias, performance bias, detection bias, attrition bias, reporting bias, and other bias. Each domain is categorized as low risk, high risk, and unclear risk. Critical appraisal of studies will be carried out independently by 2 reviewers. All the differences will be resolved through discussion with a third reviewer.

#### 2.6.3 Measures of treatment effects

Standard mean difference and 95% confidential interval will be pooled.

#### 2.6.4 Management of missing data

If any data are missing, the original data will be requested by email. If the missing data cannot be obtained, the data will be excluded from the study.

#### 2.6.5 Assessment of heterogeneity and data synthesis

First of all, the pairwise meta-analysis will be performed using RevMan 5.3. Chi-square test will be performed to measure the heterogeneity among the direct comparison results, and *I*^2^ will be used to measure the heterogeneity. If the data of the included studies do not have the statistical heterogeneity (*I*^2^ < 50%, *P* > .1), the fixed-effects model will be used for meta-analysis; otherwise, the random-effects model will be adopted.

Stata14.0 software will be used to draw an evidence network map to visualize the comparison of the intervention measures for each outcome indicator. Then, the network meta-analysis will be performed in a Bayesian framework using Markov Chain Monte Carlo methods by WinBUGS 1.4. A random-effects model will be employed because of anticipated heterogeneity. The surface under the cumulative ranking curve will be applied to rank the size effect of treatments, and the higher surface under the cumulative ranking curve value indicates a higher rank of the intervention. The node-splitting method will be used to assess the inconsistency between direct and indirect evidences.

#### 2.6.6 Assessment of reporting biases

Comparison-adjusted funnel plots will be drawn to evaluate publication bias.^[29]^

#### 2.6.7 Subgroup analysis

Subgroup analysis will be applied based on the scale type, disease severity, and type of intervention.

#### 2.6.8 Sensitivity analysis

Sensitivity analysis will be performed to test the stability of the meta-analysis using a one-by-one elimination method.

#### 2.6.9 Ethics and dissemination

The content of this article does not involve moral approval or ethical review and would be presented in print or at relevant conferences.

## 3 Discussion

So far, many non-pharmacological interventions have been available to improve anxiety, depression, and sleep disorders in patients with postoperative glaucoma. However, which non-pharmacological intervention is more effective in improving psychological problems remains unclear. Therefore, this study will collect relevant RCTs to compare the effectiveness of non-pharmacological interventions on improving anxiety, depression, and sleep disorders in patients with postoperative glaucoma surgery, which provides references in clinical practice.

## Author contributions

**Conceptualization:** Jiong Liu, Runhai Zhou.

**Data curation:** Lei Cao.

**Formal analysis:** Lei Cao.

**Funding acquisition:** Runhai Zhou.

**Investigation:** Lei Cao.

**Methodology:** Lei Cao.

**Project administration:** Runhai Zhou.

**Resources:** Guang Yang.

**Software:** Guang Yang.

**Supervision:** Runhai Zhou.

**Validation:** Guang Yang.

**Visualization:** Guang Yang.

**Writing – original draft:** Jiong Liu, Runhai Zhou.

**Writing – review & editing:** Jiong Liu, Runhai Zhou.
